# Impact of insulin resistance and microvascular ischemia on myocardial energy metabolism and cardiovascular function: pathophysiology and therapeutic approaches

**DOI:** 10.1097/XCE.0000000000000332

**Published:** 2025-04-16

**Authors:** Ariana Y. Ramirez, Elizabeth R. Doman, Kevin Sanchez, Robert J. Chilton

**Affiliations:** a Department of Cardiology, Brooke Army Medical Center; b Department of Cardiology, University of Texas Health Sciences Center, San Antonio, Texas, USA

**Keywords:** cardiac dysfunction, cardiovascular metabolism, diabetes, insulin resistance, microcirculation

## Abstract

Insulin resistance (IR) and microvascular ischemia together result in cardiovascular dysfunction by impairing the heart’s energy balance. IR in cardiomyocytes disrupts glucose metabolism, leading to energy deficits that can drive cardiac hypertrophy and heart failure. Microvascular ischemia exacerbates these effects by limiting oxygen and nutrient delivery, intensifying oxidative stress, mitochondrial dysfunction, and cell death. IR also reduces the effectiveness of vasodilators like nitroglycerin and sodium nitroprusside, exacerbating endothelial dysfunction and oxidative stress, thus impairing oxygen delivery during ischemia. This combination of IR and microvascular ischemia heightens the risk of left ventricular dysfunction and heart failure. Understanding these interactions is critical for developing targeted therapies to improve outcomes in patients with IR and ischemic heart disease. This study examines the relationship between IR, microvascular ischemia, and myocardial metabolism, with a focus on clinical management and therapeutic strategies.

## Introduction

Insulin resistance in cardiomyocytes, coupled with microvascular ischemia, creates a challenging environment for the heart. Insulin resistance in heart muscle cells diminishes their responsiveness to insulin, a hormone critical for glucose uptake and utilization [1]. This impairment in glucose metabolism leads to energy deficits and altered heart function, which can contribute to cardiac hypertrophy and heart failure over time. Microvascular ischemia, characterized by reduced blood flow through the small vessels supplying the heart, exacerbates these issues by further limiting oxygen and nutrient delivery, intensifying the energy deficits caused by insulin resistance [2].

When insulin resistance and microvascular ischemia coexist, they create a significant energy deficit in the heart. The dual stress of impaired glucose utilization due to insulin resistance and limited oxygen and nutrient supply due to microvascular ischemia leads to increased oxidative stress, mitochondrial dysfunction, and apoptosis in cardiomyocytes [3]. The underlying pathophysiology includes reduced glucose uptake, increased fatty acid oxidation, and oxidative stress, all exacerbated by microvascular ischemia’s impact on ATP production and oxygen availability [4]. Both conditions contribute to increased inflammation, further damaging the heart muscle and accelerating the progression of heart disease [5].

## Mitochondrial criticality and arrhythmogenesis in diabetes

Diabetes is associated with profound alterations in mitochondrial function, particularly in cardiac cells, where excessive oxidative stress can drive the system into a state of mitochondrial criticality. This condition is characterized by an increased sensitivity of the mitochondrial network to minor perturbations, which can initiate a cascade of electrical disturbances. As localized depolarization spreads across the mitochondrial network, it can lead to a global collapse of the mitochondrial membrane potential, synchronizing oscillatory electrical activity within cardiac myocytes and predisposing the heart to arrhythmogenesis.

Vetter *et al*. [6], in *Frontiers in Physiology*, investigated the impact of diabetes on mitochondrial criticality in cardiac cells, focusing on how oxidative stress disrupts mitochondrial homeostasis and contributes to arrhythmias. The study utilized computational modeling and experimental data to demonstrate that diabetes weakens the resilience of the mitochondrial network, making it highly susceptible to synchronized depolarization events. A key finding of the study was that diabetic mitochondria exhibit prolonged depolarization states, reducing their ability to recover and repolarize efficiently. This delayed recovery is primarily due to oxidative stress-induced damage to mitochondrial ion channels, which impairs their role in ATP production and calcium buffering. The study further demonstrated that these metabolic disturbances alter the sodium (Na⁺) and potassium (K⁺) channel dynamics, which are essential for maintaining stable cardiac excitation–contraction coupling. As a result, electrophysiological instability arises, contributing to ventricular arrhythmias and an increased risk of sudden cardiac death in diabetic patients.

Moreover, Vetter *et al*. highlighted that therapeutic interventions targeting mitochondrial stabilization – such as antioxidant treatments, mitochondrial uncouplers, and pharmacological modulators of mitochondrial permeability transition pores – could potentially mitigate these arrhythmogenic effects. By reinforcing mitochondrial resilience, these interventions may help prevent the transition from localized depolarization to widespread electrical failure, reducing the likelihood of fatal arrhythmic events in diabetic patients.

The findings from this study emphasize the crucial role of mitochondrial network stability in maintaining cardiac electrical homeostasis. They further support the growing recognition that targeting mitochondrial dysfunction in diabetes could serve as a therapeutic strategy to lower cardiovascular risk.

## Metabolic fuel sources in the insulin-resistant ischemic myocardium

Ischemic myocardium occurs when the heart muscle is deprived of adequate blood supply, often due to coronary artery blockages [7]. This condition triggers cellular injury and dysfunction due to hypoxia, or reduced oxygen supply [8]. The heart, metabolically adaptable, typically shifts to anaerobic metabolism under hypoxic conditions, leading to lactate accumulation, decreased ATP production, and impaired energy utilization [9].

Research in rodent models of myocardial ischemia and reperfusion injury has shown that an increase in branched-chain amino acids (BCAAs) is associated with worsened cardiac insulin resistance and impaired heart function [10]. Conversely, reducing BCAA availability can enhance fatty acid utilization for ATP production and reduce triglyceride accumulation in the heart, suggesting a strong connection between BCAA metabolism and cardiac function during ischemia [11]. Ischemia followed by reperfusion can also lead to reactive oxygen species (ROS) generation, contributing to oxidative stress, cellular damage, and an inflammatory response that exacerbates tissue injury [12].

BCAAs – leucine, isoleucine, and valine – play a crucial role in metabolic regulation, particularly in cardiac function during ischemia. BCAA metabolism is tightly linked to mitochondrial function and energy homeostasis in cardiomyocytes. Under ischemic conditions, the heart shifts its metabolic preference from fatty acid oxidation to glucose and amino acid metabolism to maintain ATP production. Insulin resistance, however, disrupts BCAA catabolism, leading to their accumulation and altered substrate utilization in the ischemic myocardium [13].

Studies have shown that increased plasma BCAA levels correlate with impaired cardiac efficiency and increased oxidative stress [14]. The dysregulated BCAA catabolism in insulin resistance results in the accumulation of intermediates like branched-chain keto acids, which may activate proinflammatory pathways and impair mitochondrial function. This metabolic disturbance exacerbates myocardial dysfunction during ischemia and contributes to maladaptive cardiac remodeling.

## The effect of insulin resistance on oxygen release in ischemic microcirculation

Insulin resistance significantly impacts oxygen release in ischemic microcirculation by altering several factors that influence oxygen delivery and utilization in tissues during ischemic events [15]. Impaired glucose metabolism, a hallmark of insulin resistance, limits the availability of glucose as a critical fuel source during ischemia, exacerbating cellular energy deficits and hypoxia. Furthermore, insulin resistance blunts the normal vasodilatory response [16], reducing blood flow in the microcirculation and further hindering oxygen delivery to ischemic tissues [17].

**Fig. 1:**
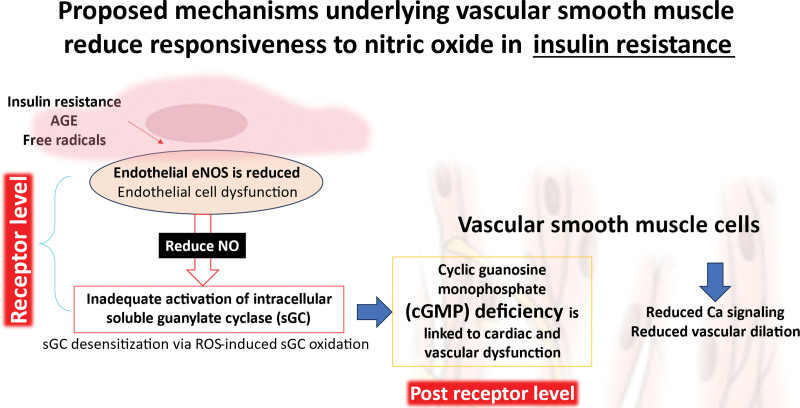
Insulin resistance leads to the release of free radicals. These free radicals result in endothelial dysfunction and reduce endothelial nitric oxide synthase (eNOS) activity and nitric oxide (NO) production. This in turn leads to vascular dysfunction through reduced guanylate cyclase activity and impaired smooth muscle relaxation.

Increased oxidative stress and inflammation associated with insulin resistance also impair endothelial function and oxygen release [18]. Elevated ROS levels damage endothelial cells, reduce nitric oxide (NO) availability, and contribute to endothelial dysfunction, all of which compromise oxygen delivery during ischemia (Fig. 1). Elevated levels of ROS can directly interact with and neutralize NO, a critical signaling molecule involved in vascular function, thereby reducing its bioavailability [19]. This interaction not only diminishes NO’s capacity to regulate endothelial cell function but also impairs its role in promoting vasodilation. As a result, the diminished NO signaling may contribute to endothelial dysfunction, leading to compromised vascular relaxation and potentially exacerbating conditions such as hypertension and atherosclerosis. Additionally, insulin resistance is linked to metabolic acidosis, which can alter oxygen release from hemoglobin through the Bohr effect [20], but this is often offset by impaired blood flow and glucose metabolism [21].

## Pathophysiology of ischemic microcirculation

Ischemic microcirculation involves impaired blood flow at the microvascular level, leading to inadequate oxygen and nutrient delivery to myocardial tissue [22]. This condition is commonly seen during percutaneous coronary interventions or acute coronary syndromes and involves endothelial dysfunction, capillary rarefaction, and inflammation, which collectively contribute to microvascular obstruction.

## Pharmacologic agents in the cardiac catheterization lab

To manage coronary ischemia, various pharmacological agents are employed to improve blood flow. Nitroglycerin, a vasodilator, acts primarily on veins by converting to NO, leading to the relaxation of vascular smooth muscle [23]. It is commonly used in the cardiac catheterization lab to alleviate coronary artery spasm and improve blood flow, particularly by dilating large epicardial vessels. This dilation indirectly enhances microcirculatory perfusion by reducing resistance in the coronary circulation and decreasing the heart’s workload.

Sodium nitroprusside, another potent vasodilator, acts on both arterial and venous smooth muscle by directly releasing NO [24]. It reduces both afterload and preload, enhancing blood flow to the coronary arteries and improving microcirculation. Its potent arterial vasodilation, however, can lead to the coronary steal phenomenon, where blood flow is diverted from ischemic regions to nonstenotic coronary arteries [25,26].

## Insulin resistance and its impact on pharmacologic agents

Insulin resistance significantly impacts the efficacy of vasodilators like nitroglycerin and sodium nitroprusside. In individuals with insulin resistance, endothelial dysfunction impairs the vasodilatory effects of these agents [27]. Although nitroglycerin and nitroprusside release NO, the oxidative stress associated with insulin resistance degrades NO [28], reducing its bioavailability and diminishing the vasodilatory response [29]. Additionally, insulin resistance may alter vascular smooth muscle cells’ responsiveness to NO, requiring higher doses of these agents for effective treatment [30]. Furthermore, conditions commonly associated with insulin resistance, such as hypertension and increased vascular stiffness, necessitate more significant intervention to achieve effective blood pressure control [31].

## Adenosine in microcirculatory management

Adenosine, a potent vasodilator, is used in the cardiac catheterization lab to assess fractional flow reserve by activating adenosine receptors, particularly A2A receptors. This activation leads to smooth muscle relaxation and enhanced blood flow, improving capillary function and oxygen delivery to tissues. Insulin resistance, however, can alter adenosine receptor function, particularly A1 and A2A receptors [32], reducing their effectiveness in promoting vasodilation, managing inflammation, and regulating glucose uptake. This alteration exacerbates vascular dysfunction and chronic inflammation, common issues in metabolic syndrome, and further complicates the management of ischemic conditions [33].

## Neural effects on microcirculation

Neural signals significantly influence microcirculation by affecting endothelial cells that line blood vessels [34]. Sympathetic and parasympathetic nerves release neurotransmitters that bind to receptors on endothelial cells, influencing vascular tone and blood flow [35]. Sympathetic stimulation generally leads to vasoconstriction, while parasympathetic activity promotes vasodilation, crucial for maintaining proper microcirculation.

## Baroreceptor and Bezold–Jarisch reflexes in cardiovascular regulation

The baroreceptor reflex, a fundamental component of the autonomic nervous system, regulates vascular tone and endothelial function by modulating sympathetic and parasympathetic outflow, maintaining stable blood pressure. The Bezold–Jarisch reflex, triggered by stimuli such as myocardial ischemia, leads to bradycardia, hypotension, and vasodilation by increasing parasympathetic activity and decreasing sympathetic activity, protecting the heart during ischemic events [36–38].

## Future directions in basic and clinical research

To advance the understanding and treatment of insulin resistance and microvascular ischemia in cardiovascular disease, research must focus on both fundamental mechanisms and translational applications. At the basic science level, investigating how insulin resistance disrupts mitochondrial function and increases oxidative stress in cardiomyocytes could lead to novel interventions targeting mitochondrial resilience. Exploring alternative metabolic pathways, such as BCAA metabolism and ketone utilization, may provide new therapeutic approaches to improve myocardial energy efficiency. Additionally, research should further examine how insulin resistance impairs endothelial NO signaling, exacerbating microcirculatory dysfunction and limiting vasodilation.

Clinically, efforts should focus on developing targeted therapies that address both metabolic and vascular dysfunction. Trials evaluating insulin-sensitizing agents, such as glucagon-like peptide-1 (GLP-1) receptor agonists and sodium-glucose cotransporter 2 (SGLT2) inhibitors, could help determine their effectiveness in improving endothelial function and reducing cardiovascular risk. Precision medicine approaches, incorporating genetic and metabolomic profiling, may allow for personalized treatment strategies tailored to individuals with distinct metabolic dysfunctions. Advances in imaging techniques, such as PET and MRI-based metabolic assessments, alongside novel biomarkers for endothelial dysfunction and mitochondrial stress, could improve early detection and intervention. By integrating these research efforts, the field can move toward more effective therapeutic strategies to mitigate the cardiovascular impact of insulin resistance and microvascular ischemia, ultimately improving patient outcomes.

## Conclusion

Insulin resistance and microvascular ischemia are closely intertwined conditions that significantly compromise cardiovascular health. Insulin resistance disrupts glucose uptake and utilization in cardiomyocytes, leading to energy deficits that contribute to the development of cardiac hypertrophy and heart failure. Microvascular ischemia exacerbates these issues by further limiting oxygen and nutrient delivery, intensifying oxidative stress and mitochondrial dysfunction, and promoting cell death.

The combined effects of insulin resistance and microvascular ischemia create a challenging environment for the heart, where the traditional metabolic flexibility is compromised, leading to increased reliance on less efficient anaerobic pathways. This not only worsens myocardial energy deficits but also diminishes the effectiveness of standard pharmacologic interventions, such as nitroglycerin and sodium nitroprusside, used to manage ischemic conditions. The altered function of adenosine receptors and the impaired vasodilatory response further complicate the management of these patients.

Given the complex interplay between insulin resistance and microvascular ischemia, a multifaceted therapeutic approach is essential. This includes improving insulin sensitivity, enhancing vascular function, and addressing oxidative stress and inflammation. By targeting these mechanisms, it may be possible to mitigate the adverse effects of these conditions and improve outcomes for patients at risk of heart failure and other cardiovascular complications.

## Acknowledgements

This study was supported by University of Texas Health Center.

### Conflicts of interest

There are no conflicts of interest.
